# Supplementary figures and images for: Reproductive barriers in cassava: Factors and implications for genetic improvement
Source: PLoS One. 2021 Nov 30;16(11):e0260576. doi: 10.1371/journal.pone.0260576 (PMC8631659; doi:10.1371/journal.pone.0260576)

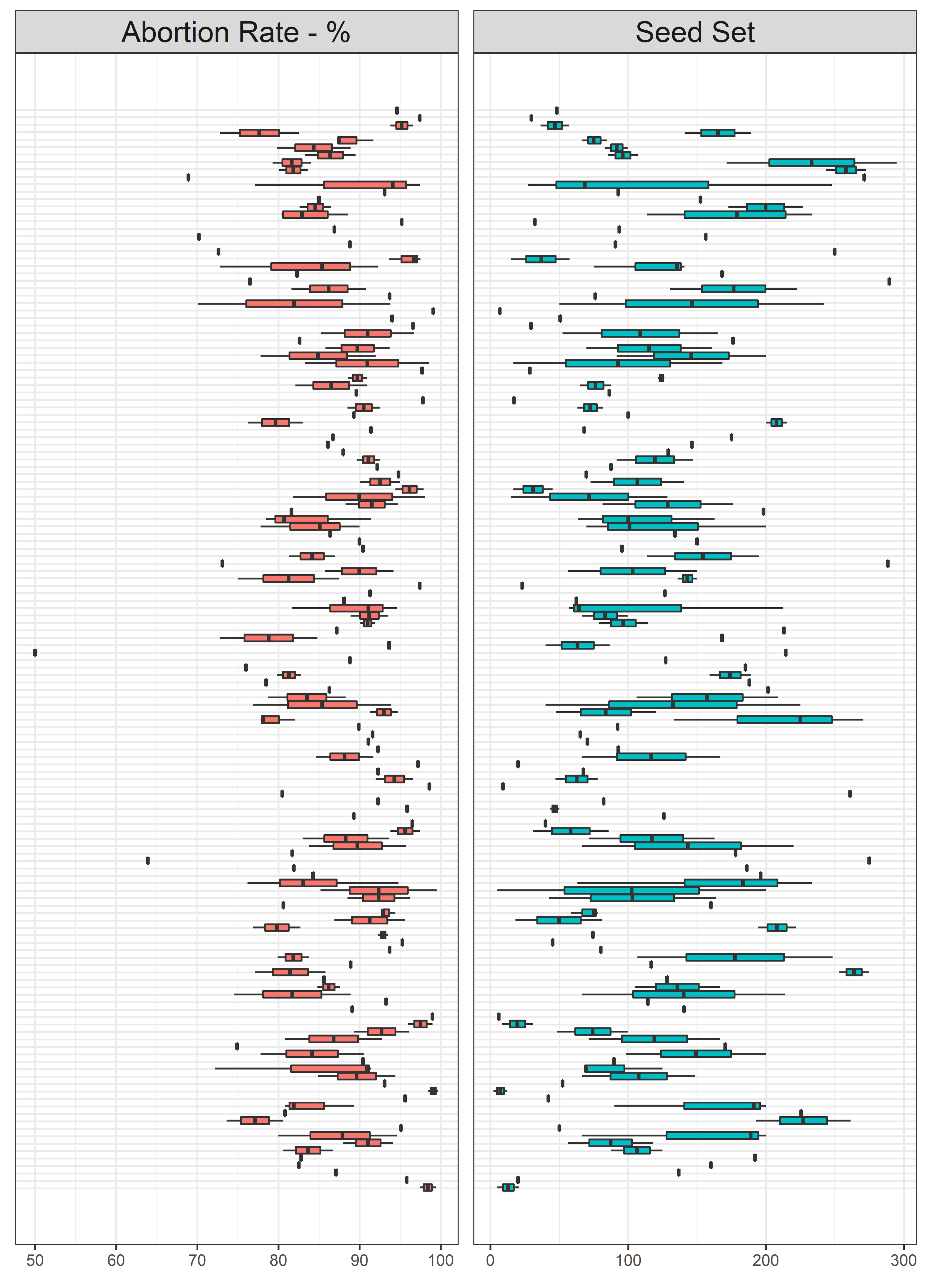

Supplement: S1 Fig — (TIF) [file pone.0260576.s001.tif]

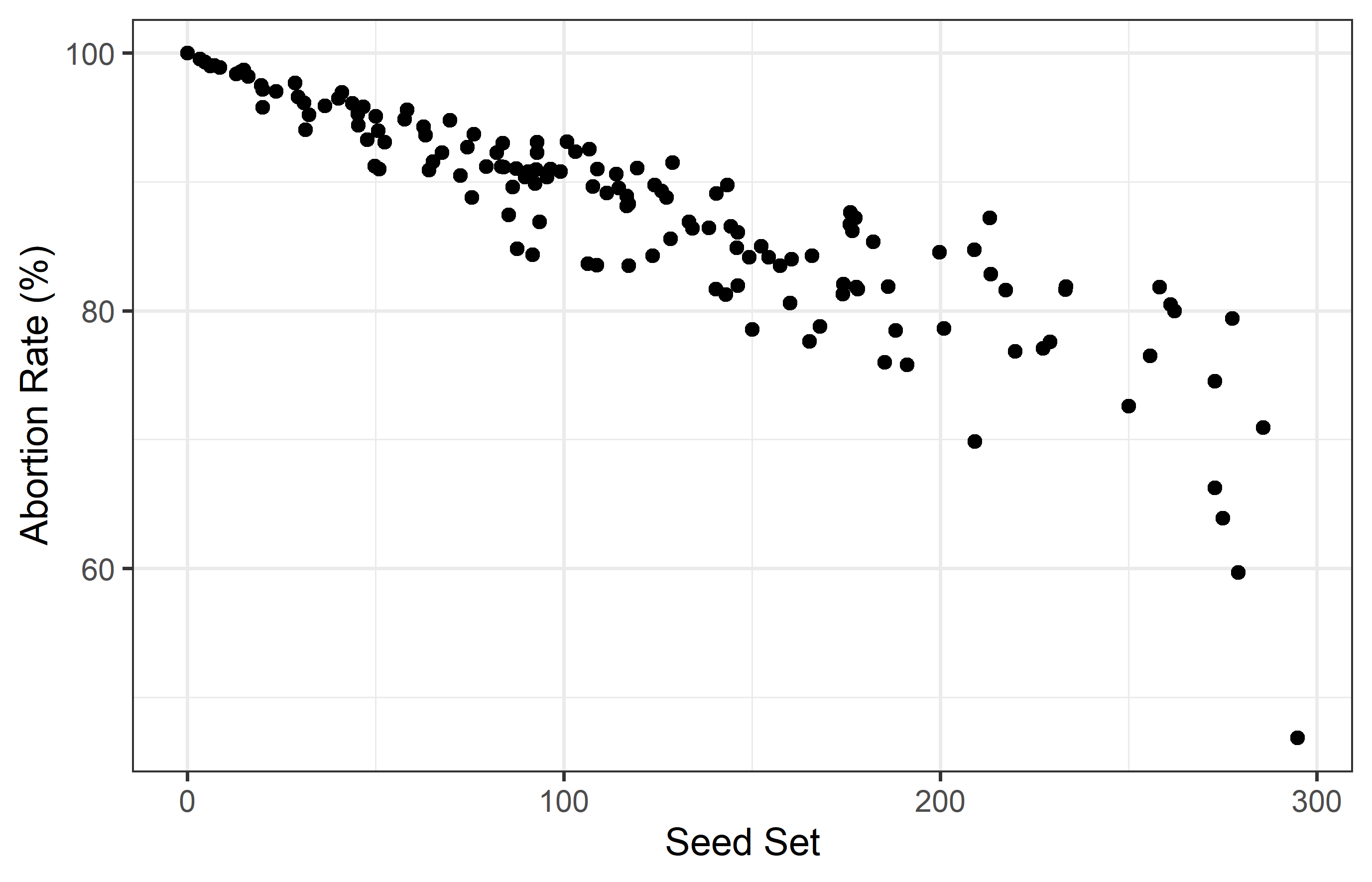

Supplement: S2 Fig — (TIF) [file pone.0260576.s002.tif]
