# Supplementary material for: Reproductive barriers in cassava: Factors and implications for genetic improvement
Source: PLoS One. 2021 Nov 30;16(11):e0260576. doi: 10.1371/journal.pone.0260576 (PMC8631659; doi:10.1371/journal.pone.0260576)
Supplement: S1 Table — (DOCX) [file pone.0260576.s003.docx]

**S1 Table**. Genotype, origin, plant shape, cyanogenic compounds (HCNs), and root color traits of the 91 cassava genotypes included in Experiments 1 and 2.

| **Genotypes** | **Types** | **Country of origin** | **State** | **Plant Shape** | **HCN** | **Root flesh color** | **Root surface color** | **Storage root cortex color** |
| --- | --- | --- | --- | --- | --- | --- | --- | --- |
| 2011-52-01 | Improved | Brazil | BA | Compact | Bitter | White | Dark brown | White/Cream |
| 2011-52-23 | Improved | Brazil | BA | Open | Bitter | White | Light brown | White/Cream |
| 2011-53-07 | Improved | Brazil | BA | Cylindrical | Intermediate | White | White/Cream | White/Cream |
| 359-09 | Improved | Brazil | BA | - | - | - | - | - |
| 7909-02 | Improved | Brazil | BA | - | - | - | - | - |
| 7909-04 | Improved | Brazil | BA | Open | - | White | Dark brown | White/Cream |
| 7909-05 | Improved | Brazil | BA | - | - | - | - | - |
| Aipim Abacate | Local variety | Brazil | BA | Open | Intermediate | White | Dark brown | White/Cream |
| Aipim Manteiga | Local variety | Brazil | PE | Compact | Intermediate | Cream | Dark brown | Pink |
| BGM-0019 | Local variety | Brazil | PA | Open | Bitter | Yellow | Yellow | Yellow |
| BGM-0046 | Local variety | Brazil | SP | Compact | Bitter | White | White/Cream | White/Cream |
| BGM-0061 | Local variety | Brazil | PA | Compact | Bitter | Yellow | Dark brown | Yellow |
| BGM-0089 | Local variety | Brazil | - | Compact | Intermediate | White | Dark brown | White/Cream |
| BGM-0093 | Local variety | Brazil | - | Open | Bitter | White | Light brown | White/Cream |
| BGM-0128 | Local variety | Brazil | MG | Open | Intermediate | White | Light brown | White/Cream |
| BGM-0131 | Local variety | Brazil | AM | Compact | Bitter | Yellow | Light brown | Yellow |
| BGM-0174 | Local variety | Brazil | - | Compact | Intermediate | White | Light brown | White/Cream |
| BGM-0216 | Improved | Brazil | RJ | Compact | Intermediate | White | Dark brown | White/Cream |
| BGM-0323 | Local variety | Colombia | Valle | Compact | Bitter | White | Dark brown | White/Cream |
| BGM-0470 | Local variety | Brazil | - | Open | Bitter | Yellow | Dark brown | Yellow |
| BGM-0661 | Local variety | Brazil | RS | Open | Bitter | Cream | Dark brown | White/Cream |
| BGM-0685 | Local variety | Brazil | MA | Open | Bitter | Cream | Dark brown | Yellow |
| BGM-0717 | Local variety | Brazil | AC | Compact | Intermediate | White | Dark brown | White/Cream |
| BGM-0728 | Improved | Brazil | BA | Compact | Bitter | Cream | Light brown | White/Cream |
| BGM-0729 | Improved | Brazil | BA | Compact | Bitter | Yellow | White/Cream | Purple |
| BGM-0816 | Local variety | Brazil | AL | Open | Sweet | White | Dark brown | Purple |
| BGM-0818 | Local variety | Brazil | SE | Open | - | White | Dark brown | White/Cream |
| BGM-0865 | Local variety | Brazil | BA | - | - | - | - | - |
| BGM-0872 | Local variety | Brazil | AP | Compact | Bitter | Yellow | Dark brown | Yellow |
| BGM-0888 | Improved | Brazil | SP | Compact | Sweet | White | Yellow | White/Cream |
| BGM-0935 | Local variety | Brazil | BA | Cylindrical | Bitter | White | Dark brown | Pink |
| BGM-0941 | Local variety | Brazil | AM | Compact | Bitter | Yellow | Light brown | Yellow |
| BGM-0942 | Local variety | Brazil | AM | Open | Bitter | Yellow | Dark brown | Yellow |
| BGM-0946 | Local variety | Brazil | AM | Compact | Bitter | Yellow | Dark brown | Yellow |
| BGM-0958 | Local variety | Brazil | AM | Cylindrical | Bitter | Yellow | Yellow | Yellow |
| BGM-0968 | Local variety | Brazil | AM | Open | Bitter | Yellow | Light brown | Purple |
| BGM-0971 | Local variety | Brazil | AM | Compact | Bitter | Yellow | Dark brown | Yellow |
| BGM-0991 | Local variety | Brazil | AM | Open | Bitter | Yellow | Yellow | Yellow |
| BGM-1023 | Local variety | Brazil | BA | Compact | Intermediate | Cream | Dark brown | Pink |
| BGM-1024 | Local variety | Brazil | BA | Umbrella | Sweet | White | Dark brown | Pink |
| BGM-1028 | Local variety | Brazil | BA | Compact | Sweet | White | Dark brown | Yellow |
| BGM-1130 | Local variety | Brazil | RN | Compact | Bitter | White | Dark brown | Pink |
| BGM-1143 | Local variety | Brazil | MT | Compact | Bitter | Yellow | Dark brown | Yellow |
| BGM-1146 | Local variety | Brazil | AM | Compact | Bitter | Yellow | Dark brown | Yellow |
| BGM-1156 | Local variety | Brazil | - | Compact | Bitter | White | Dark brown | White/Cream |
| BGM-1163 | Local variety | Brazil | - | Open | Intermediate | White | Dark brown | White/Cream |
| BGM-1174 | Local variety | Brazil | - | Umbrella | Bitter | White | Dark brown | White/Cream |
| BGM-1253 | Local variety | Nigeria | - | Compact | Intermediate | White | Dark brown | Yellow |
| BGM-1259 | Local variety | Nigeria | - | Compact | Bitter | White | Light brown | White/Cream |
| BGM-1284 | Local variety | Brazil | PE | Compact | Bitter | Yellow | Dark brown | Yellow |
| BGM-1309 | Local variety | Brazil | CE | Compact | Bitter | White | Dark brown | White/Cream |
| BGM-1332 | Local variety | Brazil | PB | Open | Sweet | White | Dark brown | Purple |
| BGM-1413 | Local variety | Brazil | RN | Open | Intermediate | White | Dark brown | Purple |
| BGM-1422 | Local variety | Brazil | RN | Open | Intermediate | White | Dark brown | Purple |
| BGM-1444 | Local variety | Brazil | PA | Open | Sweet | White | Dark brown | Purple |
| BGM-1448 | Local variety | Brazil | - | Open | Intermediate | White | Dark brown | Pink |
| BGM-1455 | Local variety | Brazil | PR | Open | Sweet | White | Dark brown | White/Cream |
| BGM-1487 | Local variety | Brazil | BA | Open | Intermediate | White | Dark brown | White/Cream |
| BGM-1583 | Local variety | Brazil | PI | Open | Bitter | White | White/Cream | Yellow |
| BGM-1638 | Local variety | Brazil | PA | Compact | Bitter | White | Dark brown | White/Cream |
| BGM-1659 | Improved | Brazil | BA | Compact | Bitter | Cream | Dark brown | Purple |
| BGM-1662 | Local variety | Brazil | PR | Open | Sweet | White | Dark brown | Purple |
| BGM-1693 | Improved | Brazil | BA | Umbrella | Bitter | White | Dark brown | Pink |
| BGM-1716 | Local variety | Brazil | AM | Open | Sweet | Cream | Dark brown | Purple |
| BGM-1760 | Local variety | Brazil | MA | Compact | Bitter | White | Dark brown | White/Cream |
| BGM-1784 | Local variety | Brazil | MA | Open | Bitter | Yellow | Light brown | Yellow |
| BGM-1811 | Improved | Brazil | BA | Compact | Bitter | White | Dark brown | White/Cream |
| BGM-1819 | Local variety | Brazil | MA | Open | Bitter | White | Dark brown | Pink |
| BGM-1942 | Local variety | Brazil | PA | Compact | Bitter | White | Yellow | White/Cream |
| BGM-2018 | Improved | Brazil | BA | Open | Intermediate | White | White/Cream | White/Cream |
| BGM-2120 | Local variety | Uganda | - | Compact | Intermediate | White | Dark brown | White/Cream |
| BGM-2127 | Local variety | Brazil | DF | Open | Bitter | Cream | Dark brown | White/Cream |
| BGM-2142 | Local variety | Brazil | PE | Compact | - | White | White/Cream | White/Cream |
| BGM-2155 | Local variety | Brazil | PE | Open | Bitter | White | White/Cream | White/Cream |
| BGM-2167 | Local variety | Brazil | ES | Compact | Sweet | Cream | Dark brown | Pink |
| BGM-2338 | Local variety | Brazil | MG | Compact | Intermediate | White | Dark brown | Pink |
| BRS Dourada | Cultivar | Brazil | BA | Compact | Intermediate | Orange | Dark brown | Pink |
| BRS Formosa | Cultivar | Brazil | BA | Compact | Bitter | White | Light brown | White/Cream |
| BRS Gema de Ovo | Cultivar | Brazil | AM | Open | Sweet | Cream | Dark brown | White/Cream |
| BRS Jari | Cultivar | Brazil | BA | Open | Intermediate | Yellow | Dark brown | Pink |
| BRS Kiriris | Cultivar | Brazil | BA | Compact | Intermediate | White | Dark brown | White/Cream |
| BRS Mulatinha | Cultivar | Brazil | BA | Compact | Bitter | White | Dark brown | White/Cream |
| BRS Novo Horizonte | Cultivar | Brazil | PE | Compact | Bitter | White | White/Cream | White/Cream |
| BRS Rosada | Cultivar | Brazil | - | Compact | Intermediate | Pink | - | - |
| BRS Tapioqueira | Cultivar | Brazil | BA | Open | Bitter | White | Light brown | White/Cream |
| BRS Verdinha | Cultivar | Brazil | BA | Open | Bitter | White | White/Cream | White/Cream |
| Cascuda | Local variety | Brazil | PR | Open | Bitter | Cream | Yellow | White/Cream |
| Cigana | Local variety | Brazil | BA | Compact | Intermediate | White | Dark brown | Yellow |
| Fécula Branca | Local variety | Brazil | PR | Compact | Intermediate | White | White/Cream | White/Cream |
| Guela de Jacú | Local variety | Brazil | PE | Open | Sweet | Cream | White/Cream | White/Cream |
| Olho Junto | Local variety | Brazil | PR | Compact | Bitter | White | Dark brown | White/Cream |
