# Supplementary material for: Reproductive barriers in cassava: Factors and implications for genetic improvement
Source: PLoS One. 2021 Nov 30;16(11):e0260576. doi: 10.1371/journal.pone.0260576 (PMC8631659; doi:10.1371/journal.pone.0260576)
Supplement: S2 Table — (DOCX) [file pone.0260576.s004.docx]

**S2 Table.** Likelihood ratio test for fixed effects and random effects for seed set and abortion rate in cassava progenies (obtained from 2016 to 2018).

| Random effects | DF | LRT | |
| --- | --- | --- | --- |
|  |  | Abortion rate (%) | Seed set |
| Male + Female vs Male | 1 | 16.41* | 11.81* |
| Female | 1 | 7.94* | 7.40* |
| Ten-days period vs Year | 1 | 7.15* | 6.81* |
| Fixed effects |  | Mean Squares | |
| Ten-days period | 35 | 88.70 | 9485.00 |
| Residual | 455 | 153.12 | 16728.75 |
| Coefficient of variation (%) |  | 14.60 | 85.59 |

DF: degrees of freedom; * significant difference at 5% probability based on an *X²* test.
