# Supplementary material for: Reproductive barriers in cassava: Factors and implications for genetic improvement
Source: PLoS One. 2021 Nov 30;16(11):e0260576. doi: 10.1371/journal.pone.0260576 (PMC8631659; doi:10.1371/journal.pone.0260576)
Supplement: S3 Table — (DOCX) [file pone.0260576.s005.docx]

**S3 Table**. Assessment of the self-pollination performed in 13 of the 21 clones (Experiment 2) to determine the number of pollen grains that adhered to the surface of the stigma (PGA), the number of pollen grains that germinated on the surface of the stigma (PGG), pollen tube growth in the pistil (PTG), and the number of fertilized ovules (NFO).

| Parent | Anthesis Period | Rep | PGA | PGG | PTG | NFO |
| --- | --- | --- | --- | --- | --- | --- |
| Aipim Abacate | Pre Anthesis | L1 | 2 | 2 | 5 | 3 |
| Aipim Abacate | Pre Anthesis | L2 | 2 | 2 | 5 | 3 |
| Aipim Abacate | Pre Anthesis | L3 | 2 | 1 | 5 | 3 |
| Aipim Abacate | Pre Anthesis | L4 | 1 | 1 | 5 | 3 |
| Aipim Abacate | Pre Anthesis | L5 | 1 | 1 | 5 | 1 |
| Aipim Abacate | Anthesis | L2 | 3 | 1 | 5 | 2 |
| Aipim Abacate | Anthesis | L3 | 3 | 1 | 5 | 1 |
| Aipim Abacate | Anthesis | L4 | 3 | 1 | 5 | 2 |
| Aipim Abacate | Anthesis | L5 | 2 | 1 | 5 | 1 |
| BGM-0019 | Pre Anthesis | L1 | 1 | 1 | 5 | 1 |
| BGM-0019 | Pre Anthesis | L2 | 1 | 1 | 5 | 1 |
| BGM-0019 | Pre Anthesis | L3 | 1 | 0 | 0 | 0 |
| BGM-0019 | Pre Anthesis | L4 | 1 | 0 | 0 | 0 |
| BGM-0019 | Anthesis | L1 | 2 | 2 | 5 | 3 |
| BGM-0019 | Anthesis | L2 | 2 | 2 | 5 | 3 |
| BGM-0019 | Anthesis | L3 | 2 | 2 | 5 | 1 |
| BGM-0661 | Anthesis | L1 | 1 | 0 | 0 | 0 |
| BGM-0661 | Anthesis | L2 | 1 | 0 | 0 | 0 |
| BGM-0661 | Anthesis | L3 | 2 | 1 | 5 | 3 |
| BGM-0661 | Anthesis | L4 | 2 | 0 | 0 | 0 |
| BGM-0661 | Post Anthesis | L1 | 2 | 1 | 5 | 3 |
| BGM-0661 | Post Anthesis | L2 | 2 | 1 | 5 | 1 |
| BGM-0685 | Pre Anthesis | L1 | 3 | 0 | 0 | 0 |
| BGM-0685 | Pre Anthesis | L2 | 3 | 0 | 0 | 0 |
| BGM-0728 | Pre Anthesis | L1 | 3 | 1 | 5 | 1 |
| BGM-0728 | Pre Anthesis | L2 | 2 | 1 | 5 | 1 |
| BGM-0728 | Pre Anthesis | L3 | 1 | 1 | 5 | 3 |
| BGM-0728 | Pre Anthesis | L4 | 1 | 1 | 5 | 3 |
| BGM-0728 | Pre Anthesis | L1 | 3 | 1 | 5 | 3 |
| BGM-0728 | Pre Anthesis | L2 | 3 | 1 | 5 | 1 |
| BGM-0728 | Pre Anthesis | L3 | 3 | 1 | 5 | 2 |
| BGM-0728 | Pre Anthesis | L4 | 2 | 0 | 0 | 0 |
| BGM-0728 | Pre Anthesis | L5 | 3 | 0 | 0 | 0 |
| BGM-1693 | Pre Anthesis | L1 | 3 | 0 | 0 | 0 |
| BGM-1693 | Pre Anthesis | L2 | 3 | 0 | 0 | 0 |
| BGM-1693 | Pre Anthesis | L3 | 2 | 0 | 0 | 0 |
| BGM-1760 | Pre Anthesis | L1 | 1 | 0 | 0 | 0 |
| BGM-1760 | Pre Anthesis | L2 | 1 | 0 | 0 | 0 |
| BGM-1760 | Pre Anthesis | L3 | 1 | 0 | 0 | 0 |
| BGM-1760 | Pre Anthesis | L4 | 1 | 0 | 0 | 0 |
| BGM-1760 | Pre Anthesis | L5 | 1 | 0 | 0 | 0 |
| BGM-1760 | Pre Anthesis | L1 | 3 | 0 | 0 | 0 |
| BGM-1760 | Pre Anthesis | L2 | 3 | 0 | 0 | 0 |
| BGM-1760 | Pre Anthesis | L3 | 3 | 0 | 0 | 0 |
| BGM-1760 | Anthesis | L1 | 2 | 0 | 0 | 0 |
| BGM-1760 | Anthesis | L2 | 2 | 1 | 5 | 2 |
| BGM-1760 | Anthesis | L3 | 1 | 1 | 5 | 1 |
| BGM-1760 | Anthesis | L4 | 1 | 1 | 5 | 3 |
| BGM-2020 | Anthesis | L1 | 1 | 1 | 5 | 3 |
| BGM-2020 | Anthesis | L2 | 2 | 0 | 0 | 0 |
| BGM-2020 | Anthesis | L3 | 2 | 1 | 5 | 3 |
| BGM-2020 | Anthesis | L4 | 2 | 1 | 5 | 3 |
| BGM-2338 | Pre Anthesis | L2 | 2 | 0 | 0 | 0 |
| BGM-2338 | Pre Anthesis | L3 | 3 | 1 | 5 | 1 |
| BGM-2338 | Pre Anthesis | L4 | 3 | 1 | 5 | 2 |
| BGM-2338 | Pre Anthesis | L5 | 3 | 1 | 5 | 2 |
| BGM-2338 | Anthesis | L1 | 1 | 1 | 5 | 2 |
| BGM-2338 | Anthesis | L2 | 1 | 0 | 0 | 0 |
| BGM-2338 | Anthesis | L3 | 1 | 1 | 5 | 3 |
| BGM-2338 | Anthesis | L4 | 1 | 1 | 5 | 1 |
| BGM-2338 | Anthesis | L5 | 1 | 1 | 5 | 2 |
| BGM-2338 | Post Anthesis | L1 | 1 | 0 | 0 | 0 |
| BGM-2338 | Post Anthesis | L2 | 1 | 0 | 0 | 0 |
| BGM-2338 | Pre Anthesis | L1 | 2 | 1 | 5 | 1 |
| BRS Dourada | Pre Anthesis | L1 | 3 | 0 | 0 | 0 |
| BRS Dourada | Pre Anthesis | L2 | 3 | 0 | 0 | 0 |
| BRS Dourada | Pre Anthesis | L3 | 3 | 0 | 0 | 0 |
| BRS Dourada | Pre Anthesis | L4 | 3 | 0 | 0 | 0 |
| BRS Dourada | Post Anthesis | L1 | 3 | 0 | 0 | 0 |
| BRS Dourada | Post Anthesis | L2 | 1 | 0 | 0 | 0 |
| BRS Dourada | Post Anthesis | L3 | 2 | 0 | 0 | 0 |
| BRS Jari | Pre Anthesis | L1 | 2 | 0 | 0 | 0 |
| BRS Jari | Pre Anthesis | L2 | 3 | 1 | 5 | 2 |
| BRS Jari | Pre Anthesis | L3 | 3 | 1 | 5 | 3 |
| BRS Jari | Anthesis | L1 | 2 | 2 | 5 | 3 |
| BRS Jari | Anthesis | L2 | 2 | 0 | 0 | 0 |
| BRS Jari | Anthesis | L3 | 3 | 1 | 5 | 1 |
| BRS Jari | Anthesis | L4 | 3 | 1 | 5 | 1 |
| BRS Jari | Anthesis | L5 | 2 | 1 | 5 | 2 |
| BRS Kiriris | Pre Anthesis | L1 | 2 | 0 | 0 | 0 |
| BRS Kiriris | Pre Anthesis | L2 | 2 | 1 | 5 | 1 |
| BRS Kiriris | Anthesis | L1 | 2 | 0 | 0 | 0 |
| BRS Kiriris | Anthesis | L2 | 2 | 0 | 0 | 0 |
| BRS Kiriris | Anthesis | L3 | 2 | 0 | 0 | 0 |
| BRS Kiriris | Post Anthesis | L1 | 2 | 0 | 0 | 0 |
| BRS Kiriris | Post Anthesis | L2 | 2 | 0 | 0 | 0 |
| BRS Kiriris | Post Anthesis | L3 | 1 | 0 | 0 | 0 |
| BRS Kiriris | Post Anthesis | L4 | 1 | 0 | 0 | 0 |
| BRS Novo Horizonte | Pre Anthesis | L1 | 1 | 0 | 0 | 0 |
| BRS Novo Horizonte | Pre Anthesis | L2 | 1 | 0 | 0 | 0 |
| BRS Novo Horizonte | Pre Anthesis | L3 | 2 | 0 | 0 | 0 |
| BRS Novo Horizonte | Pre Anthesis | L4 | 2 | 0 | 0 | 0 |
| BRS Novo Horizonte | Pre Anthesis | L5 | 1 | 0 | 0 | 0 |
| BRS Novo Horizonte | Pre Anthesis | L1 | 1 | 1 | 5 | 1 |
| BRS Novo Horizonte | Pre Anthesis | L2 | 1 | 1 | 5 | 1 |
| BRS Novo Horizonte | Anthesis | L1 | 3 | 1 | 5 | 2 |
| BRS Novo Horizonte | Anthesis | L2 | 2 | 0 | 0 | 0 |
| BRS Novo Horizonte | Anthesis | L3 | 2 | 0 | 0 | 0 |
| BRS Novo Horizonte | Anthesis | L4 | 2 | 0 | 0 | 0 |
| BRS Novo Horizonte | Anthesis | L5 | 2 | 0 | 0 | 0 |
| BRS Novo Horizonte | Post Anthesis | L2 | 3 | 0 | 0 | 0 |
| BRS Novo Horizonte | Post Anthesis | L3 | 2 | 0 | 0 | 0 |
| BRS Novo Horizonte | Post Anthesis | L4 | 2 | 0 | 0 | 0 |
