# Supplementary material for: Reproductive barriers in cassava: Factors and implications for genetic improvement
Source: PLoS One. 2021 Nov 30;16(11):e0260576. doi: 10.1371/journal.pone.0260576 (PMC8631659; doi:10.1371/journal.pone.0260576)
Supplement: S4 Table — (DOCX) [file pone.0260576.s006.docx]

**S4 Table**. Best linear unbiased predictors for the random effects of female and male parents nested to female parents for seed set characteristics, abortion rate, and the seed set of selected parents included in Experiment 2.

| Female | Male | Maternal effect | | | | Nested effect | |
| --- | --- | --- | --- | --- | --- | --- | --- |
|  |  | Abortion Rate | | Seed Set | | Abortion Rate | Seed Set |
|  |  | Value | Rank | Value | Rank |  |  |
| 2011-52-01 | BGM-0685 | 0.49 | 62 | -6.65 | 61 | 4.25 | -35.23 |
|  | BGM-2338 |  |  |  |  | -0.92 | 7.16 |
| 2011-52-23 | BGM-0685 | -0.02 | 32 | 11.60 | 11 | 3.09 | -21.92 |
|  | BGM-2338 |  |  |  |  | -3.26 | 70.87 |
| 2011-53-07 | 2011-52-01 | 0.49 | 61 | -6.03 | 59 | 1.96 | -15.28 |
|  | Aipim Manteiga |  |  |  |  | 1.19 | -14.79 |
|  | BGM-2338 |  |  |  |  | 0.16 | 4.60 |
| 7909-02 | 7909-05 | 0.78 |  | -4.38 |  | -1.10 | 5.08 |
|  | BGM-0888 |  |  |  |  | -0.34 | -1.29 |
|  | BGM-1253 |  |  |  |  | 3.34 | -9.48 |
|  | BRS Kiriris |  |  |  |  | 1.67 | 1.78 |
|  | Cascuda |  |  |  |  | 1.70 | -14.59 |
| 7909-04* | 7909-02 | -2.63 | 2 | 21.02 | 4 | -16.92 | 77.69 |
|  | Cascuda |  |  |  |  | -0.85 | 11.00 |
| 7909-05 | 7909-04 | -1.04 | 7 | 12.02 | 9 | -8.03 | 79.52 |
|  | BGM-1253 |  |  |  |  | -1.25 | -8.86 |
|  | BRS Kiriris |  |  |  |  | 2.25 | -19.95 |
| Aipim Abacate* | BRS Jari | 0.61 | 69 | -8.65 | 69 | 4.13 | -36.49 |
| Aipim Manteiga | 2011-52-23 | -0.66 | 14 | 12.38 | 8 | -2.99 | 27.91 |
|  | BGM-0685 |  |  |  |  | 0.90 | -5.57 |
|  | BRS Dourada |  |  |  |  | -0.35 | -8.81 |
|  | Cascuda |  |  |  |  | -2.00 | 38.75 |
| BGM-0019* | BGM-0685 | -0.09 | 30 | -3.66 | 48 | -0.59 | -15.46 |
| BGM-0061 | BGM-0685 | 0.41 | 56 | -8.29 | 67 | 2.74 | -34.56 |
|  | BGM-1163 |  |  |  |  | 0.04 | -0.42 |
| BGM-0089 | BGM-0685 | 0.59 | 68 | -9.96 | 77 | 3.97 | -42.02 |
| BGM-0093 | BGM-0046 | -0.25 | 25 | 7.29 | 17 | -1.71 | 30.78 |
| BGM-0128 | BGM-0661 | 0.49 | 60 | -8.86 | 70 | 3.32 | -37.39 |
| BGM-0470* | BGM-0888 | -1.19 | 5 | 7.39 | 16 | -8.05 | 31.21 |
| BGM-0661* | 7909-04 | 0.78 | 76 | -9.26 | 73 | 1.87 | -13.57 |
|  | BGM-0816 |  |  |  |  | 3.38 | -25.52 |
| BGM-0685* | BGM-0728 | -0.76 | 9 | 11.33 | 13 | -5.11 | 47.83 |
| BGM-0717 | BGM-0888 | 0.58 | 67 | -11.22 | 80 | 3.93 | -47.34 |
| BGM-0728* | BGM-0935 | -0.67 | 13 | 10.39 | 14 | -4.51 | 43.86 |
| BGM-0888 | 7909-04 | 0.12 | 44 | 5.53 | 21 | -2.89 | 22.89 |
|  | BGM-0128 |  |  |  |  | 5.49 | -22.52 |
|  | BGM-0991 |  |  |  |  | -1.82 | 22.97 |
| BGM-1693* | BRS Mulatinha | 0.35 | 52 | -3.97 | 49 | 2.36 | -16.77 |
| BGM-1716 | 7909-02 | 0.05 | 39 | -1.37 | 41 | -0.29 | -5.99 |
|  | BGM-0046 |  |  |  |  | 0.64 | 0.19 |
| BGM-1760* | 7909-02 | 0.31 | 49 | -4.92 | 54 | 2.10 | -20.74 |
| BGM-1811 | BGM-1784 | -0.20 | 27 | 0.83 | 32 | -1.39 | 3.51 |
| BGM-1819 | BGM-0968 | -0.73 | 12 | 23.24 | 3 | -4.93 | 98.08 |
| BGM-1942 | 7909-02 | 0.22 | 46 | -2.48 | 44 | 1.47 | -10.47 |
| BGM-2120* | BGM-2338 | -0.51 | 16 | 4.46 | 23 | -3.46 | 18.84 |
| BGM-2167* | BRS Tapioqueira | -0.74 | 10 | 11.50 | 12 | -4.97 | 48.54 |
| BGM-2338 | 2011-52-23 | -0.48 | 18 | 3.79 | 25 | -5.88 | 40.52 |
|  | Aipim Manteiga |  |  |  |  | -2.68 | 12.47 |
|  | BGM-0685 |  |  |  |  | 1.65 | -10.62 |
|  | Fécula Branca |  |  |  |  | 3.65 | -26.37 |
| BRS Dourada* | 7909-02 | 0.50 | 63 | -5.24 | 55 | 1.16 | -6.85 |
|  | BGM-0661 |  |  |  |  | 2.24 | -15.27 |
| BRS Gema de Ovo* | BGM-1583 | 0.70 | 75 | -7.57 | 65 | 4.70 | -31.94 |
| BRS Jari* | 7909-02 | -0.47 | 19 | 6.00 | 20 | 2.32 | -14.93 |
|  | Aipim Abacate |  |  |  |  | -5.50 | 40.25 |
| BRS Kiriris* | 7909-02 | 0.43 | 57 | -4.46 | 52 | 2.89 | -18.81 |
| BRS Mulatinha* | 7909-02 | -2.44 | 3 | 45.43 | 1 | 2.56 | -24.94 |
|  | Aipim Abacate |  |  |  |  | 4.12 | -38.26 |
|  | BGM-0888 |  |  |  |  | -2.13 | 27.12 |
|  | BGM-1156 |  |  |  |  | -4.48 | 52.26 |
|  | BGM-1332 |  |  |  |  | -5.32 | 84.27 |
|  | BGM-1487 |  |  |  |  | -8.28 | 60.62 |
|  | BGM-1716 |  |  |  |  | -2.00 | 37.92 |
|  | BGM-1784 |  |  |  |  | -7.11 | 51.49 |
|  | BGM-2018 |  |  |  |  | 6.12 | -58.74 |
| BRS Novo Horizonte* | 7909-02 | -0.52 | 15 | 9.73 | 15 | 3.57 | -22.37 |
|  | BGM-1784 |  |  |  |  | -7.07 | 63.44 |
| BRS Rosada | Fécula Branca | 0.63 | 71 | -8.18 | 66 | 4.24 | -34.52 |
| BRS Tapioqueira* | 7909-02 | -2.70 | 1 | 20.76 | 5 | -1.83 | 0.70 |
|  | 7909-04 |  |  |  |  | -1.77 | 15.24 |
|  | BGM-0661 |  |  |  |  | 0.77 | 8.31 |
|  | BGM-0818 |  |  |  |  | -0.40 | -0.42 |
|  | BGM-1253 |  |  |  |  | 2.39 | -36.39 |
|  | BGM-1332 |  |  |  |  | -4.09 | 47.90 |
|  | BRS Mulatinha |  |  |  |  | -13.33 | 52.30 |
| BRS Verdinha* | BGM-1583 | 0.06 | 41 | 1.72 | 28 | 3.31 | -23.42 |
|  | BGM-2167 |  |  |  |  | -2.91 | 30.69 |
| Cascuda | 2011-52-23 | -0.79 | 8 | 12.60 | 7 | -8.77 | 99.93 |
|  | 7909-04 |  |  |  |  | 2.34 | -20.32 |
|  | Aipim Manteiga |  |  |  |  | 0.26 | 6.69 |
|  | BGM-0685 |  |  |  |  | 0.18 | -5.90 |
|  | BGM-1259 |  |  |  |  | 1.45 | -19.59 |
|  | BRS Tapioqueira |  |  |  |  | -0.79 | -7.62 |
| Fécula Branca* | Aipim Manteiga | 0.82 | 80 | -9.62 | 75 | 5.51 | -40.59 |

A total of 86 female parents were evaluated in Experiment 1. * parents evaluated in Experiment 2.
