# Supplementary material for: Reproductive barriers in cassava: Factors and implications for genetic improvement
Source: PLoS One. 2021 Nov 30;16(11):e0260576. doi: 10.1371/journal.pone.0260576 (PMC8631659; doi:10.1371/journal.pone.0260576)
Supplement: S5 Table — (DOCX) [file pone.0260576.s007.docx]

**S5 Table**. Number of fertilized flowers for each parent included in Experiments 1 and 2.

| Female parent | Number of fertilized flowers | | Female parent | Number of fertilized flowers | |
| --- | --- | --- | --- | --- | --- |
|  | Experiment 1 | Experiment 2 |  | Experiment 1 | Experiment 2 |
| 2011-52-01 | 519 | - | BGM-1259 | 239 | - |
| 2011-52-23 | 761 | - | BGM-1284 | 93 | - |
| 2011-53-07 | 319 | - | BGM-1309 | 170 | - |
| 359-09 | 112 | - | BGM-1332 | 334 | - |
| 7909-02 | 1348 | - | BGM-1413 | 103 | - |
| 7909-04 | 385 | 27 | BGM-1422 | 37 | - |
| 7909-05 | 452 | - | BGM-1444 | 228 | - |
| Aipim Abacate | 61 | 56 | BGM-1448 | 175 | - |
| Aipim Manteiga | 737 | - | BGM-1487 | 96 | - |
| BGM-0019 | 73 | 58 | BGM-1583 | 339 | - |
| BGM-0046 | 357 | - | BGM-1638 | 61 | - |
| BGM-0061 | 258 | - | BGM-1659 | 247 | - |
| BGM-0089 | 88 | - | BGM-1662 | 243 | - |
| BGM-0093 | 223 | - | BGM-1693 | 87 | 24 |
| BGM-0128 | 174 | - | BGM-1716 | 265 | - |
| BGM-0131 | 82 | - | BGM-1760 | 166 | 68 |
| BGM-0174 | 55 | - | BGM-1784 | 220 | - |
| BGM-0323 | 347 | - | BGM-1811 | 198 | - |
| BGM-0470 | 70 | 5 | BGM-1819 | 190 | - |
| BGM-0661 | 512 | 99 | BGM-1942 | 244 | - |
| BGM-0685 | 227 | 25 | BGM-2018 | 440 | - |
| BGM-0717 | 172 | - | BGM-2120 | 61 | 25 |
| BGM-0728 | 289 | 85 | BGM-2127 | 378 | - |
| BGM-0729 | 139 | - | BGM-2142 | 121 | - |
| BGM-0816 | 996 | - | BGM-2155 | 73 | - |
| BGM-0872 | 73 | - | BGM-2167 | 119 | 11 |
| BGM-0888 | 416 | - | BGM-2338 | 491 | 63 |
| BGM-0941 | 72 | - | BRS Dourada | 175 | 28 |
| BGM-0942 | 95 | - | BRS Formosa | 83 | - |
| BGM-0946 | 80 | - | BRS Gema de Ovo | 272 | 20 |
| BGM-0958 | 88 | - | BRS Jari | 1063 | 86 |
| BGM-0968 | 128 | - | BRS Kiriris | 134 | 136 |
| BGM-0971 | 64 | - | BRS Mulatinha | 1846 | 14 |
| BGM-0991 | 556 | - | BRS Novo Horizonte | 228 | 77 |
| BGM-1023 | 630 | - | BRS Rosada | 104 | - |
| BGM-1024 | 486 | - | BRS Tapioqueira | 771 | 101 |
| BGM-1028 | 109 | - | BRS Verdinha | 559 | 3 |
| BGM-1130 | 535 | - | Cascuda | 850 | - |
| BGM-1143 | 102 | - | Cigana | 104 | - |
| BGM-1146 | 112 | - | Fecula Branca | 207 | 45 |
| BGM-1163 | 350 | - | Guela Jacu | 78 | - |
| BGM-1174 | 70 | - | Olho Junto | 32 | - |
| BGM-1253 | 875 | - |  |  |  |
