# Supplementary material for: Reproductive barriers in cassava: Factors and implications for genetic improvement
Source: PLoS One. 2021 Nov 30;16(11):e0260576. doi: 10.1371/journal.pone.0260576 (PMC8631659; doi:10.1371/journal.pone.0260576)
Supplement: S6 Table — (DOCX) [file pone.0260576.s008.docx]

**S6 Table**. Relationship between the number of pollen grains that adhered to the stigma surface (PGA) and pollen tube growth in the pistil (PTG) with the number of pollen grains that germinated on the stigma surface (PGG) during pollen tube development in the pistil for different cassava crosses.

| Traits | Classes | PGG | | | | Total |
| --- | --- | --- | --- | --- | --- | --- |
|  |  | No germinated pollen grains | 1 to 5 germinated pollen grains | 6 to 25 germinated pollen grains | 26 or more germinated pollen grains |  |
| PGA | 1 to 5 pollen grains | 369 | 138 | 15 | 1 | 523 |
|  | 6 to 25 pollen grains | 172 | 71 | 21 | - | 264 |
|  | 26 or more pollen grains | 139 | 66 | 25 | 39 | 269 |
| PTG | Pollen grains germinated on the stigma surface | - | 40 | - | - | 40 |
|  | Tip of the pollen tube in the stylet | - | 4 | - | - | 4 |
|  | Tip of the pollen tube inside the ovary | - | 10 | 3 | - | 13 |
|  | Tip of the pollen tube close to the ovary | - | 3 | 1 | - | 4 |
|  | Pollen tube penetrated the pseudomicropyle | - | 218 | 57 | 40 | 315 |
| Total |  | 680 | 275 | 61 | 40 | 1,056 |
