# Supplementary material for: Reproductive barriers in cassava: Factors and implications for genetic improvement
Source: PLoS One. 2021 Nov 30;16(11):e0260576. doi: 10.1371/journal.pone.0260576 (PMC8631659; doi:10.1371/journal.pone.0260576)
Supplement: S7 Table — (DOCX) [file pone.0260576.s009.docx]

**S7 Table**. Frequency of number of pollen grains that adhered to the stigma surface (PGA), number of pollen grains that germinated on the stigma surface (PGG), pollen tube growth in the pistil (PTG) and number of fertilized ovules (NFO) observations of the pollen tube in the cassava pistil at pre-anthesis, anthesis, and post-anthesis.

| Traits | Classes | Pre Anthesis | Anthesis | Post Anthesis | Total |
| --- | --- | --- | --- | --- | --- |
| PGA | 1 to 5 pollen grains | 214 | 179 | 130 | 523 |
|  | 6 to 25 pollen grains | 91 | 103 | 70 | 264 |
|  | 26 or more pollen grains | 107 | 98 | 64 | 269 |
| PGG | No germinated pollen grain | 268 | 246 | 166 | 680 |
|  | 1 to 5 germinated pollen grains | 97 | 104 | 74 | 275 |
|  | 6 to 25 germinated pollen grains | 32 | 22 | 7 | 61 |
|  | 26 or more germinated pollen grains | 15 | 8 | 17 | 40 |
| PTG | No pollen grain germinated on the stigma surface | 268 | 246 | 166 | 680 |
|  | Pollen grains germinated on the stigma surface | 13 | 17 | 10 | 40 |
|  | Tip of the pollen tube in the stylet | 2 | 1 | 1 | 4 |
|  | Tip of the pollen tube inside the ovary | 7 | 5 | 1 | 13 |
|  | Tip of the pollen tube close to the ovary | 1 | 2 | 1 | 4 |
|  | Pollen tube penetrated the pseudomicropyle | 121 | 109 | 85 | 315 |
| NFO | No fertilized ovules | 291 | 271 | 179 | 741 |
|  | 1 fertilized ovule | 28 | 28 | 19 | 75 |
|  | 2 fertilized ovules | 44 | 36 | 24 | 104 |
|  | 3 fertilized ovules | 49 | 45 | 42 | 136 |
| Total |  | 412 | 380 | 264 | 1,056 |
