# Supplementary material for: Reproductive barriers in cassava: Factors and implications for genetic improvement
Source: PLoS One. 2021 Nov 30;16(11):e0260576. doi: 10.1371/journal.pone.0260576 (PMC8631659; doi:10.1371/journal.pone.0260576)
Supplement: S8 Table — (DOCX) [file pone.0260576.s010.docx]

**S8 Table**. Mean test of anthesis periods and the number of pollen grains that adhered to the stigma surface (PGA), the number of pollen grains that germinated on the stigma surface (PGG), pollen tube growth (PTG), and the number of fertilized ovules (NFO) evaluated for crosses between 21 cassava parents.

| Factor | Levels | PGA | PGG | PTG | NFO |
| --- | --- | --- | --- | --- | --- |
| Anthesis Period | Pre Anthesis | 1.74 A | 0.55 AB | 1.74 AB | 0.74 AB |
|  | Anthesis | 1.79 A | 0.47 B | 1.54 B | 0.64 B |
|  | Post Anthesis | 1.86 A | 0.64 A | 1.98 A | 0.89 A |
| PGA | Low | - | 0.30 c | 1.23 c | 0.48 c |
|  | Median | - | 0.50 b | 1.74 b | 0.67 b |
|  | High | - | 0.86 a | 2.29 a | 1.11 a |

Means followed by the same capital letters (anthesis period) and lower-case letters in the columns (number of pollen grains that adhered to the surface of the stigma - PGA) did not differ significantly based on a Tukey test (p<0.05).
