# Supplementary material for: Reproductive barriers in cassava: Factors and implications for genetic improvement
Source: PLoS One. 2021 Nov 30;16(11):e0260576. doi: 10.1371/journal.pone.0260576 (PMC8631659; doi:10.1371/journal.pone.0260576)
Supplement: S9 Table — (DOCX) [file pone.0260576.s011.docx]

**S9 Table**. Heterotic groups based on a principal component analysis of the parent and female-male interaction effect (Parent/F×M) for attributes related to the reproductive abilities of different cassava genotypes.

| Parent/F×M cluster | Parent |
| --- | --- |
| 1 | BGM-0089, BGM-0093, BGM-0128, BGM-0470, BGM-0717, BGM-1690, BGM-2044, BGM-2120 and BRS Kiriris |
| 2 | BGM-0061 and BGM-0888 |
| 3 | BGM-0019, BGM-0661, BGM-0685, BGM-0728, BRS Novo Horizonte and BRS Tapioqueira |
| 4 | 2011-52-01, 2011-52-23, 7909-04, Aipim Abacate, BGM-1693, BGM-1760, BGM-1811, BGM-2167, BGM-2338, BRS Dourada, BRS Gema de Ovo, BRS Jari, BRS Mulatinha, BRS Verdinha and Fécula Branca |
