# Supplementary material for: Reproductive barriers in cassava: Factors and implications for genetic improvement
Source: PLoS One. 2021 Nov 30;16(11):e0260576. doi: 10.1371/journal.pone.0260576 (PMC8631659; doi:10.1371/journal.pone.0260576)
Supplement: S10 Table — (DOCX) [file pone.0260576.s012.docx]

**S10 Table**. Frequency observed of number of pollen grains that adhered to the stigma surface (PGA), number of pollen grains that germinated on the stigma surface (PGG), pollen tube growth in the pistil (PTG) and number of fertilized ovules (NOF) parameters between genotypes clustered based on parent and Female-male interaction (Parent/F×M) in cassava.

| Traits | Classes | Parent/F×M cluster | | | | | |
| --- | --- | --- | --- | --- | --- | --- | --- |
|  |  | 1×1 | 1×3 | 1×4 | 3×3 | 3×4 | 4×4 |
| PGA | 1 to 5 pollen grains | 2 | 60 | 72 | 59 | 222 | 108 |
|  | 6 to 25 pollen grains | 7 | 23 | 38 | 63 | 76 | 57 |
|  | 26 or more pollen grains | 0 | 42 | 11 | 64 | 114 | 38 |
| PGG | No germinated pollen grains | 8 | 85 | 104 | 95 | 262 | 126 |
|  | 1 to 5 germinated pollen grains | 1 | 30 | 17 | 57 | 102 | 68 |
|  | 6 to 25 germinated pollen grains | 0 | 8 | 0 | 19 | 25 | 9 |
|  | 26 or more germinated pollen grains | 0 | 2 | 0 | 15 | 23 | 0 |
| PTG | No pollen grains germinated on the stigma surface | 8 | 85 | 104 | 95 | 262 | 126 |
|  | Pollen grains germinated on the stigma surface | 0 | 3 | 1 | 1 | 27 | 8 |
|  | Tip of the pollen tube in the stylet | 0 | 0 | 1 | 0 | 3 | 0 |
|  | Tip of the pollen tube inside the ovary | 0 | 7 | 0 | 2 | 0 | 4 |
|  | Tip of the pollen tube close to the ovary | 0 | 1 | 1 | 2 | 0 | 0 |
|  | pollen tube penetrated the pseudomicropyle | 1 | 29 | 14 | 86 | 120 | 65 |
| NFO | No fertilized ovule | 8 | 96 | 107 | 100 | 292 | 138 |
|  | 1 fertilized ovule | 1 | 7 | 6 | 21 | 21 | 19 |
|  | 2 fertilized ovules | 0 | 8 | 8 | 24 | 36 | 28 |
|  | 3 fertilized ovules | 0 | 14 | 0 | 41 | 63 | 18 |
| Total |  | 9 | 125 | 121 | 186 | 412 | 203 |
