# Supplementary material for: Reproductive barriers in cassava: Factors and implications for genetic improvement
Source: PLoS One. 2021 Nov 30;16(11):e0260576. doi: 10.1371/journal.pone.0260576 (PMC8631659; doi:10.1371/journal.pone.0260576)
Supplement: Attachment — Submitted filename: Sousa et al_PlosOne_2020 response to reviewers v2.docx [file pone.0260576.s015.docx]

**Reviewer #1:** Additional Editor Comments**: PONE-D-21-16565.pdf**

The reviewers have expressed their satisfaction at the way manuscript has been revised, although Reviewer 1 has raised a small point, please address it.

Comment from Reviewer 1: Although I am not convinced that how a non-vital stain can be used to ascertain pollen viability. in context of the large amount of data generated based on crossing, I suggest that the authors should at least clearly mention that for performing each cross, fresh pollen were taken. Fresh pollen is likely to have viability closer to fertility of pollen.

**Response:** Thanks for your comment. Through “Material and methods” we emphasize the use of fresh pollen grains in the pollinations.

For example, in line 116-117: “Male flowers were collected during the morning of the anthesis day (7–9 am), while the crosses were performed between 9 am and 4 pm by distributing the fresh pollen grains on the stigmas”.
